# Role of apparent diffusion coefficient (ADC) and MRI-derived parameters in identifying p53-abnormal subtypes of endometrial cancer

**DOI:** 10.1016/j.ejro.2026.100776

**Published:** 2026-06-18

**Authors:** Sara Parviz, Shahrzad Sepidbar, Soheila Sarmadi, Fatemeh Nili, Fahimeh Zeinalkhani, Mohammadreza Tahamtan, Madjid Shakiba, Mahrooz Malek

**Affiliations:** a Tehran University of Medical Sciences (TUMS) Department of Radiology, Medical Imaging Center, Imam Khomeini Hospital Complex (IKHC) Musculoskeletal Imaging Center (MIRC), Iran; b Tehran University of Medical Sciences (TUMS) Department of Radiology, Medical Imaging Center, Imam Khomeini Hospital Complex (IKHC), Iran; c Department of Pathology, Yas Hospital Complex, Faculty of Medicine, Tehran University of Medical Sciences, Tehran, Iran; d Department of pathology, Tehran university of medical sciences, IKHC, Tehran, Iran; e Advanced Diagnostic and Interventional Radiology Research Center (ADIR), Department of Radiology, Imam Khomeini Hospital, Tehran University of Medical Sciences, Tehran, Iran; f Department of Radiology, Tehran University of Medical Sciences, Imam Khomeini Hospital, Tehran, Iran; g Department of Radiology, Advanced Diagnostic and Interventional Radiology Research Center, Tehran University of Medical Sciences, Tehran, Iran; h Department of Radiology, Medical Imaging Center, Imam Khomeini Hospital Complex (IKHC) Musculoskeletal Imaging Center (MIRC), Tehran University of Medical Sciences, Tehran, Iran

**Keywords:** Endometrial cancer, P53, Magnetic resonance imaging, Apparent diffusion coefficients

## Abstract

**Background:**

Non-invasive imaging techniques such as magnetic resonance imaging (MRI) offer advantages over repeated biopsies for assessing tumor characteristics, though histological and molecular analysis remain the diagnostic gold standard. Since P53 expression is an important prognostic factor linked to the histopathological patterns of endometrial cancer (EC), using MRI-derived indices to distinguish between TP53-mutated (P53abn) and non-P53abn subtypes is clinically practical. This study aimed to determine the predictive value of MRI-based markers, especially apparent diffusion coefficients (ADC), for differentiating P53abn and non-P53abn subtypes in patients with EC.

**Methods:**

This retrospective study was performed on 115 patients with known EC. All MR imaging studies were performed on 1.5 T MR imaging units. The presence of p53abn subtype was determined by immunohistochemical staining (IHC). Data were analyzed using R version 4.4.1.

**Results:**

A statistically significant difference was observed in mean ADC values between patients with and without p53abn by IHC, with notably lower ADC values in the P53abn group. However, no significant association was identified between P53abn status and other imaging parameters, such as mass T2, outer myometrium T2, or the mass-to-psoas intensity ratio. Multivariable logistic regression analysis identified ADC value and tumor’s histological subtype as the primary determinants of p53abn EC. ROC curve analysis further demonstrated that ADC measurement could effectively predict p53abn. The best cutoff point of ADC to discrimination was 695, yielding a sensitivity of 100% and specificity of 83%.

**Conclusion:**

In patients with EC, assessing tumor ADC values on MRI can serve as a valuable tool for predicting p53abn subtype.

## 1 Introduction

Endometrial cancer (EC) is one of the most common malignancies of the female reproductive system worldwide, and the morbidity and mortality from EC are increasing with a trend toward younger age [1], [2]. Evidence indicates that the occurrence and spread of EC is related not only to estrogen levels but also to tumor cell proliferation and apoptosis [3]. Maintaining a dynamic balance between cell proliferation and apoptosis is essential for normal physiological function [4], [5], disruption of this balance can lead to tumor development. Ki−67 and p53 are key markers associated with cellular proliferation and apoptosis. Among them, p53 functions as a crucial tumor suppressor gene that regulates cell cycle progression, inhibits abnormal growth, and promotes apoptosis [6], [7]. P53abn subtype is currently the only molecular marker known to predict prognosis in EC, and its presence is associated with poor clinical outcomes. Clinically, p53abn status can aid in differentiating serous from endometrioid histotypes and may act as a prognostic marker within specific histotypes or across different subtypes. Because most pathologists do not have access to p53 sequencing, p53 IHC, which is rapid, easy, and inexpensive, is used as an alternative for p53 mutation analysis. Therefore, p53 IHC is widely used in EC specimens [8], [9], [10], [11], [12].

Recently, MRI has gained attention as a noninvasive method for preoperative risk assessment in patients with EC. Diffusion-weighted MRI (DWI-MRI) suggests biochemical markers for assessing tumor microstructure by evaluating the restricted diffusion of water protons within intra- and extracellular environments. Standard in vivo DWI-MRI protocols enable the calculation of apparent diffusion coefficients (ADCs), which quantitatively reflect these diffusion characteristics [13], [14]. Studies have reported that the mean ADC value, which correlates with p53abn in esophageal squamous cell carcinoma, can serve as a noninvasive biomarker to provide prognostic information. Moreover, ADC analysis enables quantitative assessment of image characteristics—such as histogram distribution, pixel intensity, and gray-level variations—within a defined region of interest [15], [16], [17]. Although ADC analysis has been explored as a potential prognostic indicator for assessing p53abn in various cancers, limited research has specifically evaluated its predictive value in EC. Therefore, this study aimed to investigate the ability of ADC measurements to predict p53abn EC.

## 2 Materials and methods

In this retrospective study, we evaluated patients with EC who were referred to our center between September 2023 and September 2025. The inclusion criteria were histopathologically confirmed EC, availability of a pathology slide, and completion of a preoperative MRI. Exclusion criteria included other types of cancer, missing or artifact-affected pathology slides, lack of preoperative MRI, or MRI scans of insufficient quality or unsuitable for ADC assessment.

MRI of patients with a pathologically confirmed diagnosis of EC was evaluated. Before surgery, an MRI was performed on a 1.5 Tesla Siemens scanner (Magnetom Avanto). DWI was acquired using a single-shot echo-planar imaging (SS-EPI) sequence with b-values of 0 and 1000 s/mm². Acquisition parameters were included in Table 1.

**Table 1:** Summary of parameters used in the MRI protocol.

**Parameter**	**Specification**
**Scanner**	Siemens Magnetom Avanto
**Field strength**	1.5 Tesla
**Sequence**	Single-shot echo-planar imaging (SS-EPI)
**b-values**	0, 1000 s/mm²
**TR**	4800 ms
**TE**	82 ms
**Slice thickness**	4 mm
**Interslice gap**	1 mm
**Matrix size**	128 × 128
**FOV**	380 × 380 mm²
**Fat suppression**	SPAIR (spectral adiabatic inversion recovery)
**NEX**	2
**Acquisition time**	2 min 30 s

Contrast-enhanced MRI was performed following intravenous injection of gadobutrol (Gadovist®; Bayer Healthcare). The contrast agent was administered as a bolus at a dose of 0.1 mL/kg body weight. ADC maps were reconstructed from a standard diffusion-weighted imaging sequence with b-values of 0 and 1000 s/mm². Quantitative ADC measurements were performed by a junior radiologist (5 years of experience in gynecologic imaging) under the direct supervision of a senior radiologist (>10 years of experience), who reviewed all ROI placements.

Freehand polygonal regions of interest (ROIs) were placed on the solid component of each tumor. ROIs were drawn on a single representative axial slice showing the largest tumor dimension on the corresponding ADC map. ROI areas ranged from 50 to 150 mm² depending on tumor size. Cystic, necrotic, or hemorrhagic areas were identified by correlating with T2-weighted and contrast-enhanced T1-weighted images and were carefully excluded from ROI placement. Only viable, homogeneously solid tumor areas were sampled. Three repeated measurements were performed on the same slice, and the average ADC value was recorded for analysis.

Patient information, including age and sex, was also extracted from the patient records, and all data were recorded in a researcher-made checklist.

Data were analyzed using R version 4.4.1. Prior to analysis, the Shapiro–Wilk test was performed to assess normality. For continuous variables, normally distributed data were compared using the independent samples *t*-test or analysis of variance (ANOVA), while nonparametric tests (Mann–Whitney U) were applied for data not normally distributed. Categorical variables were evaluated using the chi-square test. The value of imaging-related parameters to predict P53abn subtype with the baseline characteristics (age, histological subtype, and type of tumor) was assessed by the multivariable logistic regression analysis. To assess the predictive value of ADC and other parameters, the ROC curve analysis was employed. All p-values < 0.05 were considered statistically significant.

## 3 Results

A total of 115 patients were included in the study, with a mean age of 55.2 ± 11.2 years (range: 31–80). The most prevalent tumor subtype was endometrioid carcinoma, observed in 98 patients (85.2%). Other subtypes are summarized in Table 1. The most common histologic grades were I and II, each seen in 44 patients (38.3%). The mean signal intensity (SI) of the tumor across all patients was 343.2 ± 162.0 (range: 83–1215), and the mean SI after gadolinium injection was 434.2 ± 336.9 (range: 48–1533). Ratios of tumor SI to outer myometrium SI and tumor SI to psoas SI were calculated both before and after contrast administration (Table 2).

**Table 2:** Histopathology and MRI characteristics of the study population.

**Variable**		**Mean±SD (Min-Max) or No. (%)**
**Age**		55.2 ± 11.2 (31−80)
**Histologic Subtypes**	Endometroid Carcinoma	98 (85.2)
	Serous Carcinoma	6 (5.2)
	Clear Cell Carcinoma	2 (1.7)
	Carcinosarcoma	3 (2.6)
	Endometroid and Serous Carcinoma	1 (0.9)
	Metastatic Adenocarcinoma	1 (0.9)
**Grade**	I	44 (38.3)
	II	44 (38.3)
	III	23 (20)
**Mass SI (T2)**	Pre-Contrast Injection	343.2 ± 162.0 (83–1215)
**Outer Myometrium SI (T2)**	Pre-Contrast Injection	283.7 ± 140.2 (70–1000)
**Psoas SI (T2)**	Pre-Contrast Injection	113.8 ± 59.4 (32−362)
**Mass SI**	Post-Contrast Injection	434.2 ± 336.9 (48–1533)
**Outer Myometrium SI (T2)**	Post-Contrast Injection	554.5 ± 401.9 (98–1903)
**Psoas SI**	Post-Contrast Injection	237.0 ± 172.1 (73−957)
**Mass to Outer Myometrium Ratio**	Pre-Contrast Injection	1.25 ± 0.25 (0.60–1.88)
**Mass to Psoas Ratio**	Pre-Contrast Injection	3.19 ± 0.97 (0.86–6.15)
**Mass to Outer Myometrium Ratio**	Post-Contrast Injection	0.76 ± 0.14 (0.40–1.12)
**Mass to Psoas Ratio**	Post-Contrast Injection	1.80 ± 0.70 (0.39–6.56)
**ADC**		761.6 ± 154.6 (393–1129)
**Myometrial Invasion**	More than 50%	35 (30.4% of all patients; 48.6% when excluding missing data)
	Less than 50%	32 (27.8% of all patients; 44.4% when excluding missing data)
	No	5 (4.3% of all patients; 6.9% when excluding missing data)

Regarding IHC markers, of the 115 patients in the study, P53 IHC status was available for 82 (71.3%), including 20 p53abn and 62 non-p53abn. All analyses involving P53 status were performed using complete-case analysis on these 82 patients. To assess potential bias, we compared the 33 patients with missing p53 data to the 82 patients with available data. We found no significant differences between groups in age (55.4 ± 10.6 vs 54.6 ± 12.8 years, p = 0.749), ADC (×10⁻⁶ mm²/s) values (754.5 ± 148.3 vs 778.5 ± 169.9, p = 0.484), FIGO stage distribution (p = 0.183), or histologic grade (p = 0.153). Data for other IHC indices are summarized in the supplementary material.

Tumors were classified into high-risk (grade III or histologic subtypes of serous carcinoma, carcinosarcoma, or clear cell carcinoma) and low-risk groups (grades I–II or endometrioid carcinoma). Among 110 patients with available data, 24 (21.8%) were high-risk and 86 (78.2%) were low-risk. Comparison of MRI measurements between these groups revealed that ADC was the only parameter showing a significant difference, with mean ADC (×10⁻⁶ mm²/s) values of 640.7 ± 122.6 in high-risk tumors and 803.3 ± 143.3 in low-risk tumors (P < 0.001). Mean age was also slightly higher in the high-risk group (59.5 ± 11.1 vs. 54.0 ± 11.0, P = 0.04). Correlation analysis showed that tumor risk correlated strongly with p53 status. Among the 20 p53abn patients, 15 (75%) had high-risk tumors, whereas only 6 of 61 non-P53abn patients (9.8%) were high-risk (P < 0.001, odds ratio = 27.5, 95% CI = 7.4–102.6).

Logistic regression was performed to identify factors associated with p53abn status. In the multivariable model including ADC and high-risk histologic subtype, both variables were independently associated with p53abn: ADC (OR = 0.976, 95% CI: 0.959–0.988, p = 0.0009) and high-risk histology (OR = 10.09, 95% CI: 1.27–217.6, p = 0.0545). Collinearity assessment showed no correlation (VIF = 1.000). Addition of age, FIGO stage, and tumor grade did not significantly improve model fit (likelihood ratio test, p = 0.218) nor discrimination (AUC: 0.967 vs 0.976, p = 0.429), and the simpler model was preferred based on AIC (35.8 vs 37.4). Therefore, the final model retained ADC and histologic subtype as the primary determinants of P53abn status.

**Fig. 1:**
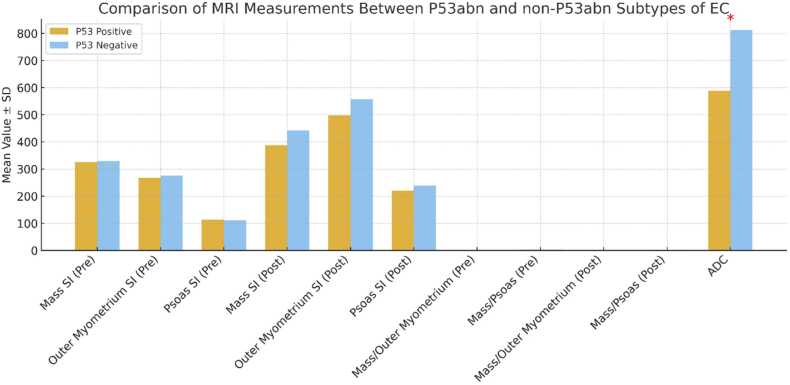
Comparison of MRI parameters between P53abn and non-P53abn endometrial cancer subtypes. Bars represent mean ± standard deviation (SD) for each parameter. Among all evaluated MRI parameters, only the ADC value demonstrated a statistically significant difference between the two groups (p < 0.001), as indicated by the red asterisk (*).

Among all MRI parameters compared between p53abn and non-p53abn patients, only ADC demonstrated a statistically significant difference. The mean ADC (×10⁻⁶ mm²/s) value was significantly lower in p53abn (588.4 ± 67.4) compared to non-P53abn patients (811.8 ± 123.2, P < 0.001) (Fig. 1).

**Fig. 2:**
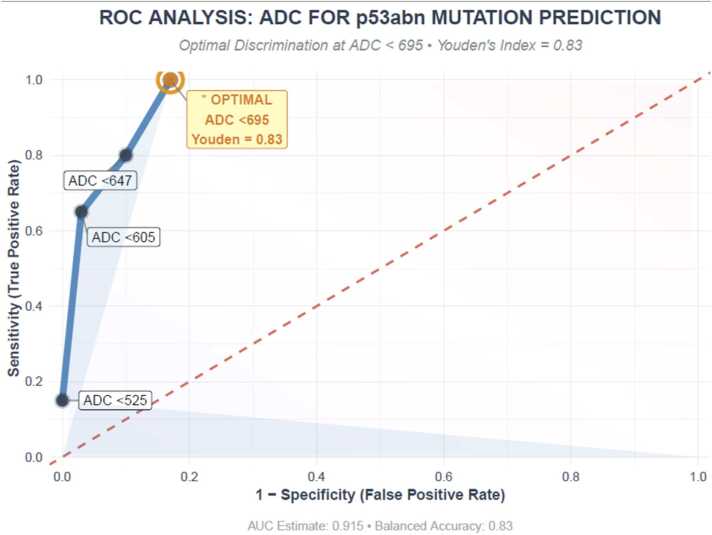
Receiver operating characteristic (ROC) curve of ADC for identifying P53abn by IHC. The area under the curve (AUC) was 0.949 (95% CI: 0.898–0.985). The optimal ADC cutoff value was < 695 × 10⁻⁶ mm²/s, yielding a sensitivity of 100% and specificity of 83%.

ROC analysis indicated that ADC was the only MRI parameter capable of suggesting p53abn status, demonstrating excellent diagnostic performance (AUC = 0.95, 95% CI: 0.90–0.99, P < 0.001). Selected ADC cutoff points and their diagnostic efficacy indices are presented in Fig. 2.

Internal validation using bootstrapping with 1000 resamples demonstrated robust diagnostic performance of the ADC cutoff < 695 for identifying p53abn EC. The optimism-corrected AUC was 0.948 (95% CI: 0.898–0.985), nearly identical to the original AUC of 0.949, indicating minimal overfitting. At the proposed cutoff of < 695, bootstrapped sensitivity was 0.95 (95% CI: 0.833–1.00) and bootstrapped specificity was 0.829 (95% CI: 0.727–0.918).

The diagnostic performance of MRI parameters for MLH1, PMS2, MSH2, and MSH6 positivity was also evaluated. For each IHC marker, the optimal MRI measurement, corresponding AUC, and selected cutoff points are summarized in supplementary material.

ROC analysis based on ADC values was also performed to identify high-risk tumors, yielding an AUC of 0.81 (95% CI: 0.71–0.92, P < 0.001). Diagnostic performance indices for this analysis are summarized in the supplementary material.

## 4 Discussion

In patients with EC, molecular classification provides prognostic information that is more reliable and independent than traditional histologic classification. This approach enables more personalized and targeted patient management. However, its primary limitation is an invasive, costly, and time-consuming process requiring tumor or blood tissue sampling [18]. Consequently, developing noninvasive methods to obtain comparable diagnostic and prognostic information holds significant clinical value. In this context, MRI and its related quantitative parameters have demonstrated considerable diagnostic and prognostic potential. In the present study, among all MRI-based parameters, ADC showed the strongest ability to distinguish p53abn and non-p53abn subtypes. P53abn tumors exhibited significantly lower ADC values, and according to ROC curve analysis, an ADC (×10⁻⁶ mm²/s) threshold of < 695 effectively differentiated p53abn and non-p53abn subtypes (Fig. 2). Our finding of an ADC cutoff < 695 × 10⁻³ mm²/s for identifying p53abn EC achieved high diagnostic performance in this cohort. To assess potential overfitting, we performed internal validation using bootstrap resampling with 1000 resamples. The optimism-corrected AUC was 0.948 (95% CI: 0.898–0.985), closely matching the original AUC of 0.949, with bootstrapped sensitivity of 0.95 (95% CI: 0.833–1.00) and specificity of 0.829 (95% CI: 0.727–0.918). These results suggest minimal overfitting within the current dataset and support the generalizability of the proposed cutoff.

Regarding missing p53 IHC data, of the 115 enrolled patients, p53 status was successfully determined in 82 (71.3%). The remaining 33 patients were excluded from the p53 subgroup analysis. To assess whether this missing data may have affected the results, we compared age, FIGO stage, histologic grade, and mean ADC values between patients with and without available p53 IHC. No significant differences were observed (all p > 0.05), suggesting that the missing data are unlikely to have introduced substantial bias (Supplementary material).

Other imaging-derived indices like Mass SI and Outer myometrium SI did not show a significant association with p53abn. The absence of a significant association with other signal intensity features may be attributed to their heightened susceptibility to confounding variables. Specifically, T2 signal intensity depends on a complex combination of tissue water content, fibrosis, and necrosis. Similarly, post-contrast enhancement is dependent on perfusion, capillary permeability, and the extracellular volume. These factors are subject to considerable inter-individual variation and can be influenced by technical parameters during image acquisition [19].

In addition to subtyping p53abn EC, we extended our analysis to discriminate between endometrioid and non-endometrioid histologies, as high-risk and low risk disease. This approach is justified by the strong association of the p53abn subtype with these clinically aggressive groups. Consistent with prior research, our findings confirm that MRI ADC value offers a powerful tool for the preoperative stratification of high-risk patients [20].

In clinical practice, molecular profiling is typically performed using tissue obtained from surgical resection or biopsy. However, noninvasive imaging offers a promising alternative for tumor characterization by identifying imaging biomarkers that correlate with molecular profiles. Although histopathological assessment remains the gold standard, MRI-based preoperative prediction of molecular subtypes could have significant clinical implications. Emerging evidence suggests that molecular classification of EC may improve patient management—for instance, by reducing undertreatment in patients with p53abn tumors.

**Fig. 3:**
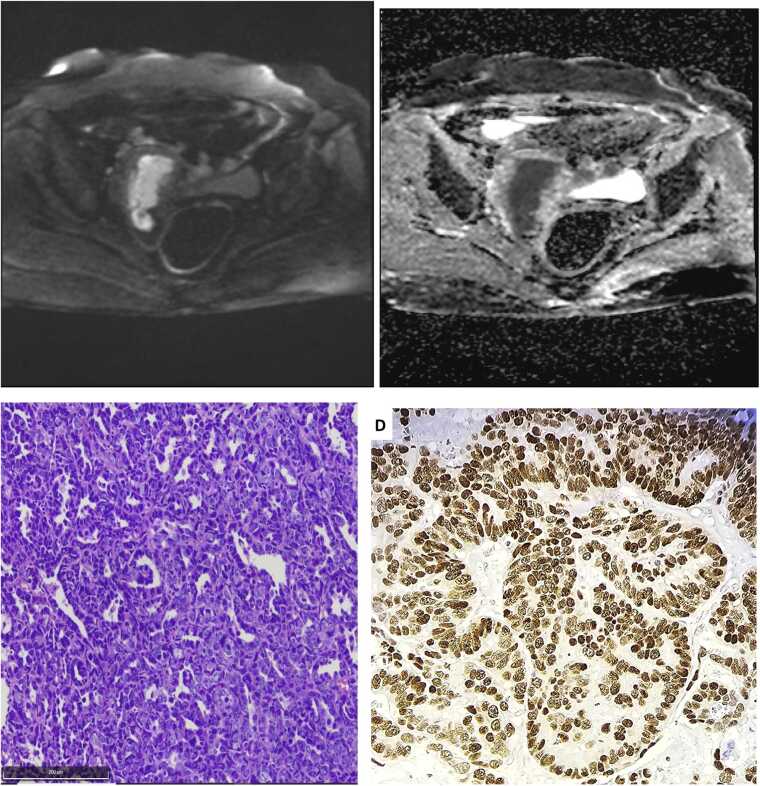
Representative case of P53abn endometrial cancer (serous carcinoma). (A) High b-value DWI (b = 1000 s/mm²) shows abnormal restricted diffusion in the endometrium (arrow). (B) Corresponding ADC map demonstrates low signal intensity with a mean ADC value of 538 × 10⁻⁶ mm²/s (arrow). (C) Histopathology (hematoxylin and eosin stain, ×400 magnification) confirms high-grade serous carcinoma. (D) Immunohistochemistry shows abnormal p53 protein expression (mutant-type pattern with diffuse strong nuclear positivity).

According to our findings, p53abn and non-p53abn subtypes may be successfully distinguished using quantitative ADC metric (Fig. 3).

**Fig. 4:**
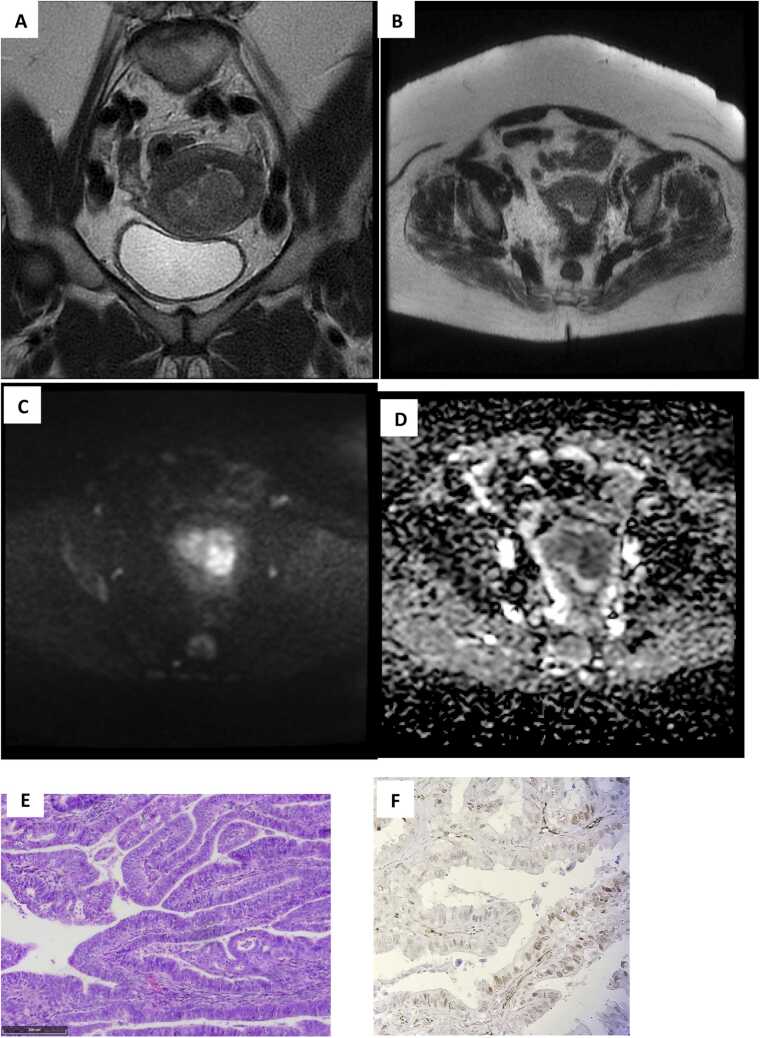
Representative case of non-P53abn endometrial cancer (low-grade endometrioid carcinoma) in a 61-year-old woman with abnormal uterine bleeding. (A, B) T2-weighted coronal and axial images demonstrate a polypoid mass within the endometrial cavity (arrows). (C) High b-value DWI (b = 1000 s/mm²) shows mild restricted diffusion (arrow). (D) Corresponding ADC map shows intermediate signal intensity with a mean ADC value of 807 × 10⁻⁶ mm²/s (arrow). (E) Histopathology (H&E stain) confirms low-grade endometrioid carcinoma (FIGO stage I). (F) Immunohistochemistry shows normal (wild-type) p53 protein expression (scattered weak nuclear positivity).

Because cancers with p53abn have a more compact arrangement of tumor cells, smaller extracellular spaces, and restricted water molecule diffusion, their ADC values were significantly lower than those of non-p53abn ECs (Fig. 4). Such a result has also been revealed in other similar studies. As recently shown by Yuying Sun et al [21], in comparison to non-p53abn ECs, p53abn ECs showed a significantly lower value of min ADC, mean ADC, P10, P50, and P90 ADC. Consequently, p53abn and non-p53abn EC may be quantitatively identified using DWI techniques, especially based on tracking ADC values. In total, multimodal MRI offers a novel method for the preoperative quantitative assessment of EC molecular typing, with potential clinical applications. Potential confounding between p53abn and high-risk histologic subtypes warrants consideration, as serous, clear cell, and carcinosarcoma subtypes are known to have a higher prevalence of p53abn [18]. Nevertheless, our collinearity assessment (VIF = 1.000) suggest that ADC and histologic subtype provide independent contributions to the model. Despite this limitation, the consistency of our findings with the existing literature supports the robustness of the identified associations.

ADC values are useful for evaluating tumor characteristics, including p53abn status. Lower ADC values, which reflect higher cellular density and restricted water diffusion, are typically associated with more aggressive tumor behavior, including higher grade, deeper myometrial invasion, and lymphovascular space invasion [22]. Our findings, consistent with prior studies, confirm that p53abn ECs have significantly lower ADC values than non-p53abn ECs. This is likely due to p53abn-driven angiogenesis and proliferation, leading to increased cellular density.

The ability to distinguish p53abn from non-p53abn EC using ADC values has several clinical implications. First, ADC measurements can help stratify patients into risk groups, facilitating treatment planning when combined with other MRI features and clinical parameters. Second, preoperative knowledge of p53 status may guide treatment decisions toward more targeted therapies. Third, lower ADC values may be associated with worse prognosis and disease-free survival in EC, particularly when integrated with other factors.

It should be noted that although ADC is a helpful marker, it's crucial to remember that other MRI methods, like T2-weighted imaging, contrast-enhanced imaging, and ADC histogram analysis, can also offer important insights into the characteristics of tumors and aid in the differentiation of molecular subtypes. In order to increase diagnostic precision and individualized treatment, research is being done to further improve the use of MRI and ADC in the diagnosis and treatment of EC. This includes integrating cutting-edge imaging methods with machine learning algorithms.

This study has several limitations that must be acknowledged. Primarily, its single-center design and relatively limited sample size limit the generalizability of the findings. Future research including larger, multi-center cohorts is warranted to validate and corroborate these results. Moreover, this study is limited by the relatively high proportion of missing P53 IHC data (28.7%). Although we found no evidence of systematic bias when comparing patients with missing versus available P53 data, the potential for unknown confounding factors cannot be completely excluded*.* Second, the manual delineation of ROIs introduces a potential source of bias, which could subsequently influence the study's conclusions. Third, the lack of inter- and intra-observer reproducibility assessment for ADC measurements is another limitation. Therefore, while the findings are promising, the strength of this study is limited and requires corroboration with a larger sample size, particularly with data acquired from 3.0 T MRI units. Future studies should incorporate radiomics, which can extract a vast number of quantitative texture features from MRI, to build even more powerful predictive models for P53 and other molecular subtypes.

## 5 Conclusion

In conclusion, our study effectively confirmed that measuring ADC value can discriminate between P53abn and non-P53abn EC. Consequently, preoperative MRI may serve as a valuable tool for guiding molecular classification of EC prior to surgery.

## CRediT authorship contribution statement

**parviz sara:** Data curation, Conceptualization. **Soheila Sarmadi:** Methodology, Investigation. **Shahrzad sepidbar:** Validation, Supervision. **malek mahrooz:** Supervision, Conceptualization. **shakiba madjid:** Project administration, Methodology. **zeinalkhani Fahimeh:** Writing – review & editing, Writing – original draft. **nili Fatemeh:** Methodology, Investigation. **Mohammadreza Tahamtan:** Writing – review & editing, Writing – original draft.

## Ethical statement

This study was conducted in accordance with the ethical standards of the institutional and/or national research committee and with the 1964 Helsinki Declaration and its later amendments. Institutional review board (IRB) approval was obtained for the use of patient data and images, and all patient identifiers were removed to ensure confidentiality. Written informed consent was obtained from all patients for publication of anonymized images and clinical information.

## Funding

This research did not receive any specific grant from funding agencies in the public, commercial, or not-for-profit sectors.

## Declaration of Competing Interest

The authors declare that they have no financial or personal relationships with individuals or organizations that could inappropriately influence (bias) their work. No conflicts of interest, such as employment, consultancies, stock ownership, honoraria, paid expert testimony, patent applications/registrations, or funding sources, are associated with this study.

This research did not receive any specific grant from funding agencies in the public, commercial, or not-for-profit sectors.

## Footnotes

Appendix A Supplementary data associated with this article can be found in the online version at doi:10.1016/j.ejro.2026.100776.
