# Supplementary material for: Role of apparent diffusion coefficient (ADC) and MRI-derived parameters in identifying p53-abnormal subtypes of endometrial cancer
Source: Eur J Radiol Open. 2026 Jun 18;17:100776. doi: 10.1016/j.ejro.2026.100776 (PMC13311172; doi:10.1016/j.ejro.2026.100776)
Supplement: Supplementary file 1 — Supplementary material [file mmc1.docx]

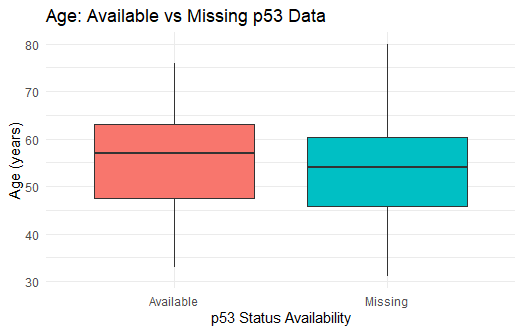

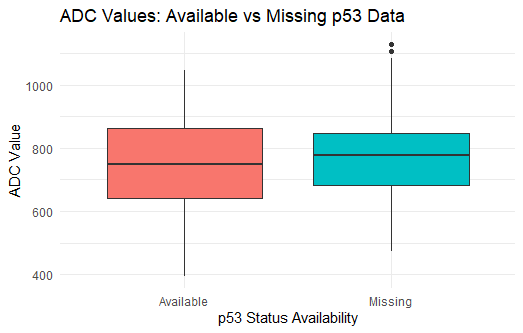


Figure S1. Comparison of age and ADC values between patients with available P53 status (n = 82) and those with missing P53 status (n = 33). Bars represent mean ± SD. No significant differences were observed between the two groups for age (55.4 ± 10.6 vs 54.6 ± 12.8 years, p = 0.749) or ADC values (754.5 ± 148.3 vs 778.5 ± 169.9 × 10⁻⁶ mm²/s, p = 0.484).

Table S1. Distribution of Different IHC Indices among Patients

| **Immunohistochemical Index** | **No. of Patients with Abnormal Finding** | **% of Patients among All Patients** | **% of Patients Setting Aside Missing Data** |
| --- | --- | --- | --- |
| **P53** | Positive: 20 | 17.4 | 24.4 |
| **MLH1** | Loss: 22 | 19.1 | 37.3 |
| **PMS2** | Loss: 23 | 20 | 26.7 |
| **MSH2** | Loss: 4 | 3.5 | 9.5 |
| **MSH6** | Loss: 8 | 7 | 9.4 |
| **PR** | Positive: 13 | 11.3 | 81.3 |
| **ER** | Positive: 34 | 29.6 | 91.9 |
|  |  |  |  |

Table S2. Selected Variables for Diagnosis of Each IHC Status and Their Cut off and Diagnostic Indices

| **Target Situation** | **Best MRI Measure** | **AUC**  **(95% CI)**  **(P-Value)** | **Selected Cut off** | **Sen.**  **(95% CI)** | **Spec.**  **(95% CI)** | **PPV**  **(95% CI)** | **NPV**  **(95% CI)** | **PLR**  **(95% CI)** | **NLR**  **(95% CI)** | **Accuracy**  **(95% CI)** |
| --- | --- | --- | --- | --- | --- | --- | --- | --- | --- | --- |
| **Loss of MLH1** | **Mass SI**  **Post Contrast** | 0.73  (0.59-0.88)  (0.003) | >380  (Youden) | 0.55  (0.32-0.76) | 0.89  (0.74-0.97) | 0.75  (0.48-0.93) | 0.76  (0.61-0.88) | 4.9  (1.8-13.3) | 2  (1.2-3.1) | 0.76  (0.63-0.86) |
| **Loss of MSH2** | **Mass SI**  **Post Contrast** | 0.82  (0.6-1)  (0.035) | >335  (Youden) | 0.75  (0.19-0.99) | 0.89  (0.75-0.97) | 0.43  (0.10-0.82) | 0.97  (0.85-0.99) | 6.9  (2.3-20.5) | 3.6  (0.65-19.5) | 0.88  (0.75-0.97) |
| **Loss of MSH6** | **ADC** | 0.79  (0.63-0.96)  (0.007) | ≥860  (Youden) | 0.75  (0.35-0.97) | 0.78  (0.67-0.87) | 0.27  (0.11-0.50) | 0.97  (0.88-0.99) | 3.5  (1.9-6.3) | 3.1  (0.94-10.5) | 0.78  (0.68-0.86) |
|  |  |  |  |  |  |  |  |  |  |  |

Table S3. Diagnostic Indices of ADC in the diagnosis of High-Risk Tumors in Different Cut off Points.

| **ADC**  **Cut off** | **TP** | **FN** | **TN** | **FP** | **Sen.**  **(95% CI)** | **Spec.**  **(95% CI)** | **PPV**  **(95% CI)** | **NPV**  **(95% CI)** | **PLR**  **(95% CI)** | **NLR**  **(95% CI)** | **Accuracy**  **(95% CI)** |
| --- | --- | --- | --- | --- | --- | --- | --- | --- | --- | --- | --- |
| **<950** | 24 | 0 | 12 | 70 | 1  (0.86-1) | 0.15  (0.08-0.24) | 0.26  (0.17-0.36) | 1  (0.74-1) | 5.8  (3.3-10.2) | ---- | 01.2  (1.1-1.3) |
| **<750** | 21 | 3 | 52 | 30 | 0.88  (0.68-0.97) | 0.63  (0.52-0.74) | 0.41  (0.28-0.56) | 0.95  (0.85-0.99) | 2.4  (1.7-3.3) | 5.1  (1.7-14.8) | 0.69  (0.59-0.78) |
| **<680**  **(Youden)** | 18 | 6 | 66 | 16 | 0.75  (0.53-0.90) | 0.80  (0.70-0.88) | 0.53  (0.35-0.70) | 0.92  (0.83-0.97) | 3.8  (2.3-6.3) | 3.2  (1.6-6.5) | 0.79  (0.70-0.87) |
| **<603** | 11 | 13 | 78 | 4 | 0.46  (0.26-0.67) | 0.95  (0.88-0.99) | 0.73  (0.44-0.92) | 0.86  (0.77-0.92) | 9.4  (3.3-26.9) | 1.8  (1.2-2.5) | 0.84  (0.76-0.90) |
